# Mechanical Cues Affect Migration and Invasion of Cells From Three Different Directions

**DOI:** 10.3389/fcell.2020.583226

**Published:** 2020-09-17

**Authors:** Claudia Tanja Mierke

**Affiliations:** Faculty of Physics and Earth Science, Peter Debye Institute of Soft Matter Physics, Biological Physics Division, University of Leipzig, Leipzig, Germany

**Keywords:** cell mechanics, confinement, extracellular matrix, inhomogeneities, elasticity, viscosity, cancer cells, stiffness

## Abstract

Cell migration and invasion is a key driving factor for providing essential cellular functions under physiological conditions or the malignant progression of tumors following downward the metastatic cascade. Although there has been plentiful of molecules identified to support the migration and invasion of cells, the mechanical aspects have not yet been explored in a combined and systematic manner. In addition, the cellular environment has been classically and frequently assumed to be homogeneous for reasons of simplicity. However, motility assays have led to various models for migration covering only some aspects and supporting factors that in some cases also include mechanical factors. Instead of specific models, in this review, a more or less holistic model for cell motility in 3D is envisioned covering all these different aspects with a special emphasis on the mechanical cues from a biophysical perspective. After introducing the mechanical aspects of cell migration and invasion and presenting the heterogeneity of extracellular matrices, the three distinct directions of cell motility focusing on the mechanical aspects are presented. These three different directions are as follows: firstly, the commonly used invasion tests using structural and structure-based mechanical environmental signals; secondly, the mechano-invasion assay, in which cells are studied by mechanical forces to migrate and invade; and thirdly, cell mechanics, including cytoskeletal and nuclear mechanics, to influence cell migration and invasion. Since the interaction between the cell and the microenvironment is bi-directional in these assays, these should be accounted in migration and invasion approaches focusing on the mechanical aspects. Beyond this, there is also the interaction between the cytoskeleton of the cell and its other compartments, such as the cell nucleus. In specific, a three-element approach is presented for addressing the effect of mechanics on cell migration and invasion by including the effect of the mechano-phenotype of the cytoskeleton, nucleus and the cell’s microenvironment into the analysis. In precise terms, the combination of these three research approaches including experimental techniques seems to be promising for revealing bi-directional impacts of mechanical alterations of the cellular microenvironment on cells and internal mechanical fluctuations or changes of cells on the surroundings. Finally, different approaches are discussed and thereby a model for the broad impact of mechanics on cell migration and invasion is evolved.

## Introduction to Cell Migration and Invasion

Cellular motility is a crucial task in many physiological functions, such as wound healing processes after tissue injury, and pathological functions. The pathological functions encompass, for instance, the migration and invasion of a malignant subset of cancer cells out of the primary tumor during cancer metastasis or the entire invasive growth mode of the primary tumor mass switching toward an aggressive and invasive state. Cancer metastasis represents a multistep consecutive process that is precisely regulated by specific biochemical and mechanical cues of the nearby microenvironment of the cancer cell. Tugging forces evoked by the migration and invasion of cells through the extracellular matrix network can be produced *in vitro* and combined with a classical 3D cell invasion assay, where the cells are plated on top of these matrices, which is termed mechano-invasion assay. It has been shown that these tugging forces induced and further promoted the invasion of cells into these matrices (Menon and Beningo, 2011). By using this assay, it was possible to reveal mechanisms regulating the invasion of cells due to randomly applied tugging on the fibers of the extracellular matrix network.

Among these genes fostering the invasiveness of cancer cells are those needed for governing of the activity of protrusive structures, such as invadopodia, in cancer cells. As these genes are elevated, it is proposed that invadopodia are assembled, whose activity is increased due to mechanical probing (Alexander et al., 2008; Albiges-Rizo et al., 2009).

In line with the hypothesis that decreased adhesion causes an enhancement in the invasive capacity of a cell, it has been detected that a wide variety of cell adhesion genes were diminished in their expression rather than increasingly expressed. In specific, the expression of ITGB3, the integrin β3 receptor subunit, has been seen to be decreased due to mechanical stimulation. In agreement with the expression results, the overexpression of the integrin β3 impaired the increase in invasion that commonly takes place after the stimulation, and thereby provides another proof that integrin β3 needs to be decreased in its expression due to mechanical cues to facilitate the cellular reaction toward the mechanical probing.

As fibronectin is necessary for accessing the mechanical stimulus through a process referred to as mechanosensing (Menon and Beningo, 2011), it seems to be likely that downregulation of integrin β3 is evoked by a regulatory feedback mechanism, which is triggered through the process of mechanosensing via the integrin β3. Apart from it, it is possible that another integrin, such as integrin β1 can take over this function and facilitate the downregulation through crosstalk associated mechanism. Nevertheless, it was intriguing that a well-known mechanoreceptor is downregulated due to mechanical probing.

**FIGURE 1:**
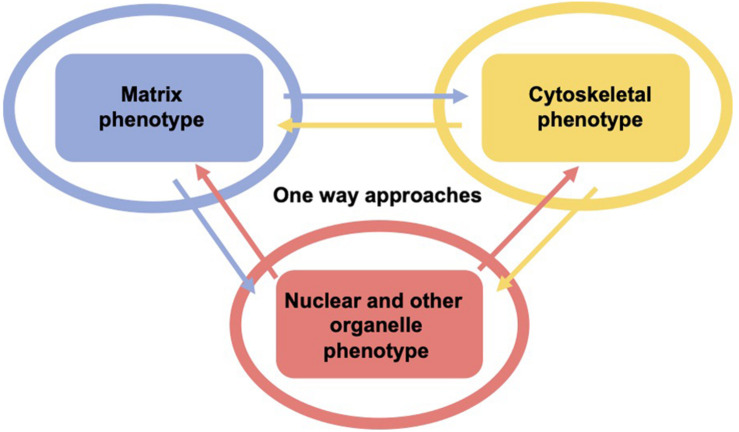
One-way approaches are utilized to analyze the three major mechanical properties including surrounding matrix, the cell’s cytoskeleton and its major the organelle the nucleus and other organelles. Hence, there are the cytoskeletal mechano-phenotype and the nuclear and other organelle mechano-phenotype. There is only one mechanical phenotype at a time that alters the matrix (blue), the cytoskeleton (yellow), and the cell nucleus and other organelles (red) in this type of approach, which is referred to as one-way approach, such as in the majority of classical migration and invasion assay.

**FIGURE 2:**
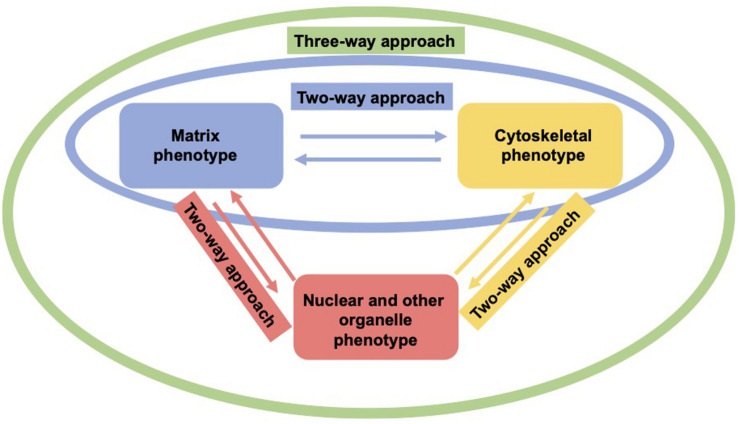
Two-way or three-approaches are an improved method for investigating the three major mechanical properties. In a two-way approach two mechanical phenotypes are altered to reveal the impact in both directions (red, yellow, and green). In a three-way approach, all three mechanical phenotypes are altered to uncover their interdependence (green).

As living cells and tissue are no static arrangement of building blocks withstanding all external mechanical cues, it is necessary to address alterations of cells and tissues during the process of cell migration and invasion through extracellular matrix environments. Consequently, the migration and invasion of cells is generally subject to ongoing fluctuations due to alterations of the mechanical cytoskeleton-based phenotype of cells, the matrix mechanical phenotype, encompassing composition, concentration, cross-linking, and degree of homogeneity, and the nuclear and other organelle mechanical phenotype, which plays a crucial role in narrow confinements restricting the movements at cellular length scale. However, an issue that has been excluded for a long time, was the inhomogeneity of extracellular matrix scaffolds affecting the migratory capacity of cells. Commonly, the mechanics-based approaches include mostly only a one-way based task focusing on the alteration of one specific phenotype in revealing some insights into the mechanisms of cell migration and invasion (Wolf et al., 2007; Fisher et al., 2009; Figure 1). Thereby, only one of the three major mechanical phenotypes, such as the matrix phenotype, the cytoskeletal phenotype, and the nuclear and other organelle phenotype are changed and its impact on at least one other phenotype is determined. More holistic two-way or three-way approaches may be promising that elucidate the interaction between cells and their microenvironment from different ways based on different directions, since there are still discrepancies between different approaches, such as the impact of cell stiffness/deformability (Figure 2; Guck et al., 2005; Mierke et al., 2011a,b; Mierke, 2019a), forces (Mierke et al., 2008; Koch et al., 2012; Kai et al., 2016), or mechanical linkages within cellular compartments and the surrounding extracellular matrix (Destaing et al., 2014; Kim and Wirtz, 2015; Martino et al., 2018; Fischer et al., 2020). Thereby, at least two phenotypes are altered to investigate the impact on the others in both directions and reveal a more complex regulatory phenomenon. The migration modes employed by cells grow steadily (Mierke, 2020), which therefore can be attributed to the growing number of biophysical techniques. However, these biophysical techniques enable the novel two-ways and three-way approaches. In line with this, a contradictory hypothesis is put forward which challenges the well-known general hypothesis that cell stiffness/deformability generally has the same effect on cell migration and invasion (Mierke, 2019a). Since cancer cell types differ largely in their biochemical and genetic phenotypes, it cannot be generally assumed, for example, that increased deformability of cells leads to increased migration and invasion by confined 3D scaffolding structures of the extracellular matrix (Jonietz, 2012; Alibert et al., 2017). Instead, one can rather hypothesize that the differences between the different cell types have different effects on the mechanical phenotype that promotes or hinders migration in interaction with other cell type-specific characteristics. Consequently, it cannot be assumed that a general and cell type-independent increased cell deformability generally causes increased migration and invasion of 3D matrix inclusions.

## Dimensionality and Heterogeneity of Microenvironments Employed for Cell Motility Analysis

On 2D substrates, cells sense, adhere to, and generate force toward a single surface mostly homogenous substrate. In 3D microenvironments the surroundings differ vastly in topology, rigidity, and uniformity form the 2D situation (Caswell and Zech, 2018). The heterogeneity of extracellular matrix mechanical cues seems to be based on dynamic cellular interactions and matrix remodeling processes evoked by cells (Malandrino et al., 2018). Primarily cancer cells and stroma cells contribute to the matrix remodeling events. This causes the extracellular matrix to become a complex mechanical issue (Malandrino et al., 2018). Tissues are commonly comprised of extracellular matrix proteins, various types of cells, blood-fueled vascular regions, in addition to a collection of signal transduction proteins for intercellular communications. When determining the structural architectures and functions of extracellular matrices, it seems promising to have a complete list of these constituents, including a list of all proteins in each intended matrix and an even more comprehensive list of all proteins that influence or process matrices in certain situations, which is referred to as a matrisome (Hynes and Naba, 2012).

Apart from these common features, the proportions within the different anatomical spaces vary considerably. On the one hand there are typically unique mechanical properties of specific tissue types (Akhtar et al., 2011). Inside tissues the architecture of them is dictated by the function (Hynes and Naba, 2012). On the other hand, tissues can look quite inhomogeneous on smaller length scales, where cells experience and perceive mechanical property changes within tissue regions (Khadpekar et al., 2019). There is also a special type of movement in cell collections referred to as asmataxis. In asmataxis, cooperative migration is transferred due to inhomogeneity, resulting in a long-ranged self-patterning of the cells, such as skeletal myoblast C2C12 cells (Khadpekar et al., 2019). Asmataxis fulfills a functional role in the spatial multicellular patterning of tissues during the physiological development of tissues or in artificial tissue engineering. Similarly, asmataxis can play a crucial task in disease states such as the progression of malignant cancer. In specific to promote the patterning of whole tissues, a reaction-diffusion model has been proposed (Turing, 1952). In line with this many diffusion-based patterning mechanisms have been envisioned and shown to be relevant in this process (Heller and Fuchs, 2015). Apart from diffusion, mechanical cues also perform a critical role in the formation of patterned tissues (Krieg et al., 2008; Shen et al., 2008; Mammoto and Ingber, 2010; Poh et al., 2014). Cells have been demonstrated to align due to topographical cues (Charest et al., 2007; Soleas et al., 2015), shear stress (Choi et al., 2014; Ostrowski et al., 2014), and cyclic strain (Liu et al., 2008; Greiner et al., 2013). The alignment of cells can be thoroughly controlled by the varying stiffness of the underlying substrate (Suk et al., 2012).

In engineered materials, the mechanical properties of the matrix are regulated by its microstructure. At exactly the same structural length scale in biomaterials, the composition and architecture differ widely among various tissue types, inside individual tissues and crucially also with the chronological age of the tissue and the development of diseases, such as cancer, acute or chronic inflammations (Akhtar et al., 2011).

Apart from structural mechanical cues, the fluid flow through the environment of the extracellular matrix can also alter the behavior of the embedded cells to align with the fluid flow (Malek and Izumo, 1996; Ng and Swartz, 2003; Miteva et al., 2010; Poduri et al., 2017). Moreover, it has been demonstrated that cells can sense the variations of tissue rigidities through the focal adhesion (FA) protein talin, which is engaged to cell-matrix receptors, such as integrins and subsequently, it is recruited to nascent FAs (Austen et al., 2015).

All in all, the heterogeneity of tissues on a cellular length scale, such as inhomogeneities of the extracellular matrix scaffold has been ignored in many bioengineered migration and invasion assays, when analyzing the cells in a 3D microenvironment. In addition, the dynamical remodeling of the microenvironment of cells has often been excluded from motility examinations of invading cells. The mechanical probing of extracellular matrices has just become a novel research focus, since the tensile stress acting on extracellular matrices supports the induction and enhancement of cell invasion. Finally, it has also been observed to have an effect on the function of the cells and the entire tissue, and even to contribute to a profound reshaping of tissues after injury during the healing process or to the differentiation of cells into tissues.

## Cell Protrusions in Migration and Invasion

Cell migration through 3D microenvironments depends on cellular contractile forces and is regulated by the substrate rigidity (Lo et al., 2000; Liu et al., 2008; Mierke et al., 2008, 2011b). There are at least two completely different structures with which cells are confronted on their route of cell migration and invasion through tissues. Firstly, the basement membranes, which assemble thin sheet like structures to anchor cells, including epithelial and endothelial cells, and subsequently to enclose entire tissues or organs. The basement membrane protects these tissues or organs from the underlying interstitial extracellular matrix, by forming a complex 3D structural architecture that is characterized by fibrillar collagens and has pores of varying sizes that allow the cells to enter/penetrate tissue or organs. Against this background, it is obviously not surprising that cells have the capacity to accommodate a wide variety of distinct migration modes in a 3D matrix (Mierke, 2019b, 2020), which either include the morphological appearance of protrusions/proturberances or even provide a mechanism to perform the specific migration mode (Friedl and Alexander, 2011). Within extracellular 3D matrices, cells exhibit a considerable amount of plasticity and are capable of alternating the migration mode in dependence of intrinsic and extrinsic parameters (Friedl and Alexander, 2011). The capability of cells to migrate and penetrate within collective sheets or strands further increases the intricacy of migration characteristics (Mayor and Etienne-Manneville, 2016). However, the focus of this review is on the individual migration and invasion of cells including the specific mechanisms of actin-driven protrusion formation. However, it seems probable that the mechanisms of protrusion at the leading edge are apparently the same as those found in leading cells in collectively migrating groups of cells. Invasive cells can interact with the underlying tissue, whereby they insert protrusive structures, such as invadopodia. Cells can move through and penetrate 3D microenvironments using at least two different major modes, the protrusion-based mode or the blebbing-based mode involving membrane detached rounded membrane bubbles (Mierke et al., 2018).

### Caveolae Serve as Mechanosensitive Membrane Invaginations

The transitions between different shapes of cells and their various functions requires plasticity and a specific part of this specialization takes place at the cell’s plasma membrane. An intrinsic characteristic of the plasma membrane of mammalian cells is their plasticity, a property essential for the perception and transduction of signals and for the capacity to cope with tension alterations caused by their environment or their own biomechanical characteristics. The caveolae are membrane invaginations that are mechanosensitive and are coupled to actin filaments (Röhlich and Allison, 1976; Singer, 1979; Valentich et al., 1997). Caveolae constitute unique invaginated membrane nanodomains that are key players in the organization of signal transduction, the homeostasis of lipids and the accommodation to membranous tension. Caveolin 1 has been identified as a major compound of caveolae (Rothberg et al., 1992), which is co-localized with the CD24 that supports actin-based cell migration in 3D collagen matrices through elevated contractile forces (Mierke et al., 2011a). Since caveolae are commonly linked to actin stress fibers, they contribute to membrane tension and the shape of cells.

The plasticity of the organization of caveolae is based on three different forms: flattened caveolae, single caveolae and clustered caveolae (Parton et al., 1994; Kiss and Botos, 2009). Caveolae are not evenly distributed in the several types of mammalian cells. They are very common in mechanically challenged cells including adipocytes, endothelial cells, fibroblasts, and muscle cells, but are practically non-existent in neurons and lymphocytes (Nassoy and Lamaze, 2012; Parton and del Pozo, 2013). It has been shown that caveolae are crucial for the regulation of multiple cascades of signaling, including several mechanisms of mechanotransduction (Parton and del Pozo, 2013). Caveolae perform a pivotal role in lipid homeostasis, and the deficiency of caveolar components leads to lipodystrophy in both mouse models and humans (Pilch and Liu, 2011). In addition, caveolae are involved in the buffering of mechanical stress at the plasma membrane (Sinha et al., 2011), what might possibly understand the occurrence of muscular dystrophies, myopathies and cardiopathies in mice and humans with mutations in caveolar components, since muscle cells are permanently subjected to mechanical stress across their plasma membrane (Galbiati et al., 2001; Garg and Agarwal, 2008; Rajab et al., 2010).

The unique feature of caveolae, its physical connection and functional interaction with the actin cytoskeleton, and particularly with the stress fibers. Since mechanically stressed cells are capable of accommodating the mechanical alterations of their microenvironment, cells should restructure their plasma membrane and actin cytoskeleton. The connection of caveolae with tension fibers can guarantee the coupling and communication between these two mechanosensors, the actin cytoskeleton and the plasma membrane, which is necessary to sustain plasma membrane integrity and to guarantee proper signaling in reply to physical signals (Echarri and Del Pozo, 2015). For example, in matrix-directed cell migration, the membrane tension is used to pull back the rear end of the migrating cell (Hetmanski et al., 2019). Invasive cancer cells traveling along the 3D cell-derived matrix of fibrillar collagen and fibronectin (Cukierman et al., 2001) can track the topology of the fibrils with defined lamellipodial and filopodial projections at the cell’s leading edge (Paul et al., 2015a; Caswell and Zech, 2018), similar to cells moving on 2D substrates. The rear retraction zone of the cell is rounded and swiftly displaced with a limited number of small retraction fibers, and adhesion complexes (Hetmanski et al., 2019). Without a chemotactic gradient, the direction of migration along matrix fibrils is assumed to be provided by the physical characteristics of the matrix, such as the strain stiffening of the matrix (Van Helvert and Friedl, 2016). Thereby, the polarization of the cells is maintained that is needed for the guided directional movement.

### Invadosomes Encompass Invadopodia and Podosomes

Generally, to migrate and invade tissues efficiently, cells need to explore and sense their microenvironmental properties, such as matrix properties or fluid flow. Thereby, they need to adapt their own cytoskeletal, nuclear, organelle, or plasma membrane mechanics and alter their surrounding matrix confinement by degradation, secretion of matrix cross-linking molecules, or exert forces to apply tensile stress on their environment. In the following, the matrix environment in *in vitro* cell culture assays can be modified to promote the migration and invasion of cells, such as cancer cells or fibroblasts. Since transient mechanical stress can provoke the maturation of invadopodia and consequently promote their migration and invasion through 3D confinements, it is necessary to understand how the mechanotransduction can signal from the outside in the cells to finally facilitate the motility of cells in 3D (first direction: structure-based apporach). These invadopodia can be analyzed in greater depths by either altering the cytoskeletal mechanical characteristics, the mechanical linkage between cellular organelles, such as the nucleus or mitochondria or by probing the surrounding extracellular matrix scaffold directly with mechanical load (second direction, extrinsic mechanics-based approach) or indirectly via the alteration of the structural cues within the matrix scaffold (third direction, cell mechanics-based approach).

Invadosomes can be seen as mechano-adhesive structures involved in the regulation of the invasive capacity of cells through their ability to breakdown the extracellular matrix (Albiges-Rizo et al., 2009; Linder, 2009; Destaing et al., 2011; Murphy and Courtneidge, 2011; Saltel et al., 2011; Boateng et al., 2012). The new term invadosome has been defined to cover the term invadopodia in cancer cells and podosomes in untransformed cells, comprising dendritic cells, endothelial cells, macrophages, osteoclasts and vascular smooth muscle cells (Marchisio et al., 1987; Gaidano et al., 1990; Kanehisa et al., 1990; Miyauchi et al., 1990; Hai et al., 2002; Moreau et al., 2003; Burns and Thrasher, 2004; Destaing et al., 2014). From a historical perspective, the terms podosome and invadopodia characterize exactly the same structure, which in epithelial cells and fibroblasts is determined by the expression of a constitutively active variant of the tyrosine kinase Src (Tarone et al., 1985; Chen, 1989). However, invadopodia and podosomes still exhibit fine variations in molecular content, dynamics and structure (Destaing et al., 2014).

Invadosomes represent hot-spots of intensified actin polymerization. The invadosome structure is comprised of two continuously polymerizing actin arrangements represented by long pillars of tightly bundled F-actin filaments aligned perpendicular to the substrate. This structure is referred to as the heart of the invadosome structure, in which an actin cluster is formed, consisting of radial F-actin filaments parallel to the substrate (Luxenburg et al., 2007; Winograd-Katz et al., 2011). Consequently, a single invadosome is identified by a dense F-actin core covered by a closed ring of adhesion molecules that colocalize on the actin cluster cloud. Multiple extracellular matrix receptors, including CD44, β1, β3, and β5 integrins, have been detected in invadosomes (Zambonin-Zallone et al., 1989; Jurdic et al., 2006; Destaing et al., 2010; Schmidt et al., 2011). Within these invadosomes, the associated receptors can sustain the localization of multiple adaptor proteins including those identified in FAs encompassing tyrosine kinases, such as focal adhesion kinase (FAK), Pyk2, and Src, small GTPases. Among these small GTPases are Cdc42, Rac and Rho, and adaptor molecules, such as p130Cas, paxillin, and vinculin. In the end, these invadosomes essentially perform two main tasks, such as exerting actin-rich and adhesive cellular protrusions or components and governing polarized secretory signaling pathways that manage the precisely regulated supply of metalloproteases required for the degradation of the extracellular matrix.

### Coupling Functions of Src and Its Substrates Between Actin-Based Adhesion and Matrix Breakdown

c-Src belongs to the family of tyrosine kinases that possess generally a distinct domain structure, encompassing a myristoylated N-terminal domain that directs Src toward membranes, two Src-homology-protein-binding domains (SH2 and SH3), and a catalytic domain shared by tyrosine-kinases. The activation of Src is facilitated through the impairment of the intramolecular interactions of SH2 with the phosphorylated Tyr527, when specific phosphatases targeting this Tyr residue and thereby causing an “opening” of the entire protein (Roskoski, 2004). Apart from its kinase activity, Src performs an adaptor function that controls the liberation of its SH2 and SH3 domains, which can interact with a variety of molecules. Similar to integrins, the alteration of c-Src activity is facilitated through conformational states, which are referred to as on “open” and “closed” conformational state due to the intracellular governance. Within the invadosome, the oscillation of conformational changes of Src controls the cellular functions. In specific detail, a constitutive active Src mutant, termed SrcY527F, has been expressed in Src-, Yes-, and Fyn-deficient (SYF) fibroblasts (Kelley et al., 2010), where it can induce the protrusion of invadosomes in a wide variety of different cell types. However, this type of invadosome is not capable of breaking down the surrounding extracellular matrix. This result indicates that the cycling of the Src activation cycle seems to be a fundamental and necessary mechanism for linking the acto-adhesive system and the breakdown activity of the extracellular matrix.

The targeted molecules of Src contribute also to this linking mechanism. Cortactin represents a Src substrate that may perform a function in the maturation of invadosomes, whereby it controls the secretory release of matrix-metalloproteinases (MMPs) within invadopodia (Clark et al., 2007; Clark and Weaver, 2008; Buschman et al., 2009). The localization of cortactin to predicted future spots of the breakdown of the extracellular matrix occurs prior to the trafficking of proteases (Artym et al., 2006). There are two novel substrates of the Src kinase, termed Tks4 and Tks5, which were characterized to function in an essential manner in the organization of invadosomes (Courtneidge, 2012). Beyond their function in the dynamics of PIP2 and the five SH3 domains that facilitate scaffolding activity, Tks4 and Tsk5 are components of the Nox3 protein complex involved in ROS synthesis, which is required for the breakdown activity of invadosomes. The functional connection between Tks4 and Tsk5 is not yet clearly elucidated, however, it may be rather obvious that they fulfill crucial tasks in governing of the linkage of the acto-adhesive system and the breakdown activity toward the extracellular matrix environment. The expression of Tks5 in Tks4^–/–^ mouse embryonic fibroblasts (MEFs), which express an active Src mutant, referred to as SrcY527F, can sufficiently rescue the normal assembly of invadosomes without the capacity to breakdown the extracellular matrix. Apart from the ratio between Tks5/Tks4, the phosphorylation of Tks4 is supposed to be crucial for the governance of this linkage. In fact, the expression of the Tks4Y25/373/508F triple mutant rescues the sustained generation of the invadosome in Tks4^–/–^ MEFs, whereas their breakdown activity is not restored (Buschman et al., 2009; Branch et al., 2012). These data suggest a regime in which the linkage between the acto-adhesive mechanism and the breakdown rate of the extracellular matrix relies on the molecular dynamics of invadosome governors. Up to now, it is assumed that the linkage of these two functionalities is exclusively controlled by mutants that alter the adaptive cycle of conformational modification or the phosphorylation dynamics. Therefore, the coupling of these two processes strongly relies on accurate cycling of the activation/inhibition of the principal invadosome governors. However, there may be differences between invadopodia and podosomes (Yamaguchi et al., 2005; Oikawa et al., 2008, Artym et al., 2011). Thus, it has been speculated that there exists a maturation process enabling a transition between the two protrusive states (Destaing et al., 2014).

### Filopodia

There is much evidence that elevated activity of lamellipodia favored increased 3D migration, invasion and metastasis. In addition, there is ample evidence that lamellipodial regulators, which comprise the compounds of the race activator Tiam-1 and the WAVE complex, are attenuated in metastatic cancer (Malliri et al., 2002; Silva et al., 2009; Sowalsky et al., 2015; Vaughan et al., 2015). It is probable, therefore, that other types of F-actin-based protrusions can supplement or counterbalance the migration into 3D. Filopodia fulfill various tasks in migrating cells, such as detecting and adapting to the chemical and physical environment, establishment of cell–cell adhesions when closing epithelial membranes with zippers and developing protrusions (Mattila and Lappalainen, 2008). Similarly, the exertion of filopodia has also been associated with the cancer invasion and the malignant progression of cancer encompassing metastasis. Fascin, which belongs to the actin bundling proteins that encourage filopodial exertion, is increasingly expressed in a variety of metastatic tumors in mice and humans (Vignjevic et al., 2007; Tan et al., 2013; Li et al., 2014; Schoumacher et al., 2014; Huang et al., 2015). In addition, the expression of myosin X is initiated by the expression of the gain-of-function mutant p53 to facilitate metastasis in mouse models of pancreatic cancer and to be implicated in the poor performance in breast cancer (Arjonen et al., 2014).

Bone morphogenetic protein (BMP) signaling has been found to stimulate the expression of ARHGEF9b in tip endothelial cells to activate Cdc42 and produce filopodia through formin like 3 (FMNL3; Wakayama et al., 2015). Filopodia through formin like 3 was also involved in angiogenesis in mammalian tissues, pointing to a conserved mechanistic pathway. Fascin fulfills a crucial task in the bundling of F-actin within filopodia in cancer and additionally induces the exertion of filopodia in tip cells of the endothelium. However, fascin also impacts the process of angiogenesis in a moderate manner (Ma et al., 2013), which highlights its functional role as a filopodial regulator that performs more redundant tasks in this specific cell type.

There are different types of filopodia, such as long filopodia-like protrusions. It has been seen that a small amount of long filopodia-like protrusions occurs near the periphery of breast cancer cells as they begin to move into the lung parenchyma and interstitium-like environments (Shibue et al., 2012). Long filopodia-like protrusions initiate the connection to the extracellular matrix in metastatic breast cancer cells through the coupled interaction of the RhoGTPase-formin axis, such as Rif-mDia2, and the integrin signaling axis, such as ILK-Parvin-Pix-Cdc42-PAK-cofilin, to prolong the lifetime of the long filopodia-like protrusions, which drives the formation of adhesions and the generation of proliferative signals through FAK–ERK signaling, subsequently supporting tumorigenesis (Shibue et al., 2012, 2013).

Filopodia can also foster the migration and invasion of cancer cells in the extracellular matrix environment. In specific, the local co-trafficking of α5β1 integrins and receptor tyrosine kinases (RTKs), encompassing the epidermal growth factor receptor 1, provide the interaction between cell–matrix adhesion receptors and RTKs (Paul et al., 2015a) and thereby repress the activity of Rac. However, they induce the activation of RhoA at the frontline of the cell’s leading edge to protrude actin-spike protrusions at the front edge of the invading cancer cells (Jacquemet et al., 2013). Due to the activation of RhoA actin-spike protrusions are created in breast and lung carcinoma cell lines that express the gain-of-function mutant p53. These specific protuberances are clearly distinguishable from lamellipodia as they do not have dendritic actin sheaths and consist of numerous small filopodia that emerge in the direction of migration in cells moving in extracellular 3D matrices and in *in vivo* tissues (Paul et al., 2015a,b). Filopodial actin spikes necessitate the formin FHOD3 that is activated through the phosphorylation downstream of the RhoA–ROCK elements. The density and structural organization of filopodia inside these protrusions lead to the hypothesis that they fulfill a crucial function in the generation of protrusive forces.

## First Direction (Structure-Based Approach): Commonly Employed Invasion Assays Where Structural and Structural-Based Mechanical Environmental Cues Are Altered

**FIGURE 3:**
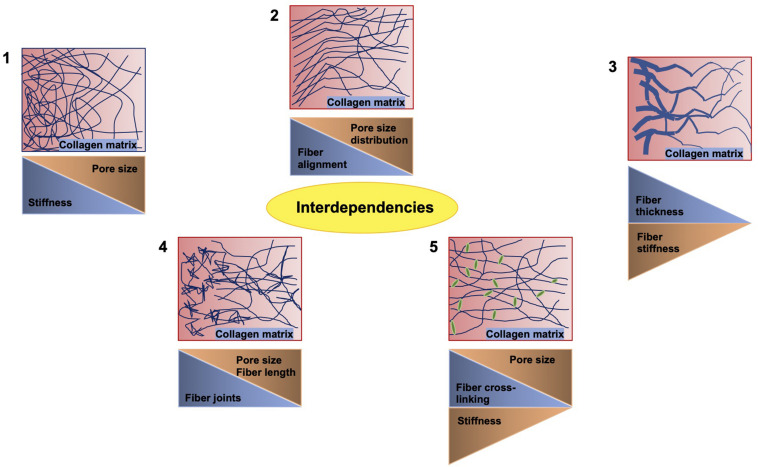
Crucial interdependencies between structural and mechanical cues of 3D collagen fiber matrices. When the stiffness of the matrix is altered, the pore size is inversely altered **(1)**. When the fiber alignment is modulated, the pore size distribution is affected **(2)**. When the fiber thickness is varied, the stiffness of the fiber is altered accordingly **(3)**. When inhomogeneities, such as joints, are increased, the fiber length and pore size are decreased **(4)**. Similarly, the cross-linking of the fibers reduces the pore size, but decreases the stiffness **(5)**.

There exists a number of studies that deal with varying concentrations of extracellular matrix proteins polymerizing into a fibrillar scaffold suitable for the migration and invasion of multiple cell types (Figure 3; Mierke et al., 2011b; Wolf et al., 2013; van Helvert et al., 2018; Anguiano et al., 2020; Le et al., 2020). When one parameter is altered usually the others may be changed accordingly, which has to be taken into account or it needs to be omitted by adapting the experimental approach. There are some examples of such interdependencies provided in Figure 3. The most commonly employed matrices are hydrogels, which constitute polymeric fibrous networks capable of retaining a large amount of water or any liquid withholding up to 95–99% of its weight. They are biomimetic in nature, as their high-water contents and diffusive transport characteristics are closely related to that of natural extracellular matrix (Spiller et al., 2011). Most hydrogels are even biocompatible, like those made with natural polymers such as agarose, alginate, chitosan, collagen, dextran, fibrin, gelatin, hyaluronic acid (HA), Matrigel, and silk (Vega et al., 2017) and those obtained from synthetic gels on the basis of polyacrylic acid (PAA), poly(ethylene glycol) (PEG), poly(hydroxyethyl methacrylate) (PHEMA), poly(vinyl alcohol) (PVA), and poly(propylene fumarate) (Hunt et al., 2014). Another feature of hydrogels that is extremely beneficial in the field of tumor environments and regenerative medicine is their strong functionalization capacity, which enables them to be easily adapted for enhanced cell adhesion and mechanical characteristics or continuous release of growth factors, cytokines including chemokines and pharmaceuticals (Buwalda et al., 2017).

### Hydrogel Concentration

The invasion of cancer cells turned out to follow a biphasic response toward the stiffness of the extracellular matrix microenvironment (Ahmadzadeh et al., 2017). The risk of breast cancer is 4–6 times higher when mammographic density is elevated (Boyd et al., 1998, 2001; McCormack and dos Santos Silva, 2006), whereby mammographic density becomes a major independent risk parameter for breast cancer (Boyd et al., 1998, 2002; McCormack and dos Santos Silva, 2006). This rise in mass density is associated with a considerably elevated accumulation of extracellular matrix proteins, especially collagen I (Guo et al., 2001; Boyd et al., 2002; Li et al., 2005), which is partially accountable for the general rise in stiffness in mammary tumors (Paszek et al., 2005; Lopez et al., 2011). Rigidity of matrix has been proven to encourage a malignant phenotype in cancer cells (Paszek et al., 2005; Alexander et al., 2008; Provenzano et al., 2009; Tilghman et al., 2010), increases the migration and invasion (Pelham and Wang, 1997; Lo et al., 2000; Kostic et al., 2009; Menon and Beningo, 2011), and impacts the intracellular signaling, pathways, causing consequently elevated proliferation (Wozniak et al., 2004; Klein et al., 2009; Provenzano et al., 2009). Even though it is obvious that matrix stiffness is a major determinant of tumor progression, the mechanisms whereby cells react to alterations in matrix stiffness are not yet fully comprehended. In parallel to the abundance of collagen, the alignment of the collagen fibers seems to have a decisive function in the progression of tumors. Changes in the arrangement and orientation of collagen fibers have been characterized and tumor-associated collagen signatures (TACS) have been recognized (Riching et al., 2014), which become apparent in a predictable manner throughout the progression of the tumor. In specific, the accumulation of aligned collagen fibers oriented perpendicular to the tumor margin, which are referred to as TACS-3, establishes motorways on which cancer cells are monitored to travel *in vivo* (Provenzano et al., 2006), and is associated with enhanced invasion and metastasis in murine models (Provenzano et al., 2008).

### Hydrogel Composition

The nanostructure of the extracellular matrix and specific collection of the extracellular matrix molecules is precisely guided in a tissue-specific manner during tissue development to provide proper functions of cells and entire organs (Smith et al., 2017). Alterations of the composition in extracellular matrix scaffold and the mechanical phenotype are observed in the course of the progression of most degenerative diseases and show the outcome of aging or, as a compensatory effort of the tissue, maintain its proper function (Kim et al., 2000; Parker et al., 2014; Klaas et al., 2016). At present, these alterations in compliance of the extracellular matrix are regarded as prognostically valuable for solid tumors (Calvo et al., 2013; Hayashi and Iwata, 2015; Reid et al., 2017).

**FIGURE 4:**
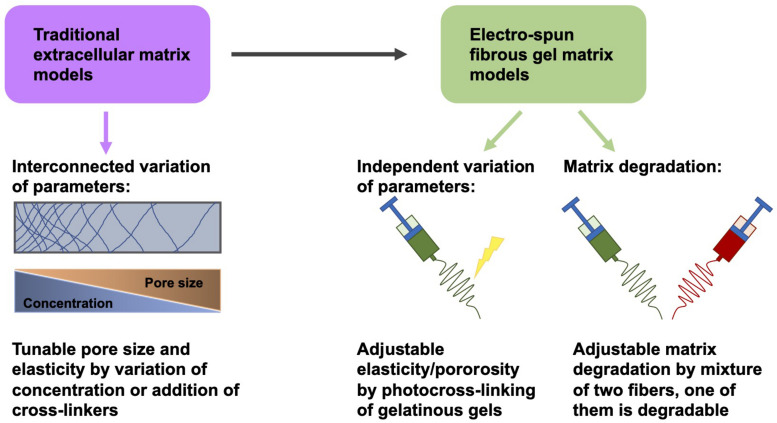
Variation of matrix parameters independently of one another. Instead of traditional fibrous matrix gels, electrospun fibrous gels are employed to obtain gels whose elasticity/porosity can be adapted independently of other parameters, such as pore size and concentration.

Knowledge of the function of mechanical and structural elements within the extracellular matrix may constitute an indispensable foundation for cancer therapy (Karuppuswamy et al., 2014). However, due to the interlinked alterations of these factors in traditional extracellular matrix model schemes, it is commonly challenging to separately distinguish these distinct parameters (Jiang et al., 2015; Hwang et al., 2016; Jun et al., 2018; Huang et al., 2020). To overcome this difficulty, an electrospun fibrous gel matrix, adjustable for elasticity/porosity, was designed, consisting of photocrosslinked gelatinous microfibers (chemical gels crosslinked in the nanometer range) with highly controlled binding, such as fiber-bound gels in the tenth of a micrometer range (Gurave et al., 2020; Huang et al., 2020) (Figure 4). This arrangement permits an independent manipulation of the microscopic fiber elasticity and porosity of the matrix, in other words the mechanical and structural requirements of the extracellular matrix can be adjusted independently. The elasticity of the fibers was coordinated with photocrosslinking parameters (Huang et al., 2020). The porosity of the material was modified by adjusting the level of inter-fiber cohesion (Figure 4). The interference of these elements of the fibrous gel matrix on the migratory capacity of tumorigenic MDA-MB-231 cells and non-tumorigenic MCF-10A cells have been quantitatively examined. MDA-MB-231 cells exhibited the strongest level of MMP-independent invasion into the matrix, which consisted of fibers with a Young’s modulus of 20 kPa and a small level of interfiber cohesion, whereas MCF-10A cells under the identical matrix circumstances demonstrated non-invasive performance (Huang et al., 2020). In addition, there are also biodegradable polymers that can be generated through electrospinning (Kai et al., 2014; Jiang et al., 2015).

There exists vastly divers types of hydrogels: firstly, physical or reversible gels in which the framework of the polymer network is achieved by physical cross-linkages such as micellar crystallites, helix formation, hydrogen bonds or hydrophobic forces, that can be “broken” (reversed) by altering the conditions of the solution, such as pH, temperature, salt concentration, and ionic strength (Caló and Khutoryanskiy, 2015), and secondly, chemical or permanent-type gels in which the cross-linked polymers are attached covalently. An essential feature for cartilage tissue engineering is the fact that hydrogels possess adaptable mechanical characteristics that are connected to the level of cross-linking and are determined by the existence and amount of the charge. Charged hydrogels modify their hydration in dependence of the pH level and their geometry when they are subjected to the electromagnetic field (Takahashi et al., 2014).

### Special Relevance of Collagen Type I for Cancer Cell Migration and Invasion

Several global transcriptomic or proteomic studies revealed special candidates that foster the onset of colon carcinogenesis due to elevated or reduced expression levels, however, among them are those additionally altered in locally improved or metastatic colorectal cancer (Langlois et al., 2014; Naba et al., 2014; Yuzhalin et al., 2018a). In specific, the proteomics analysis of the detergent-insoluble portions of paired primary tumors of the colon and liver metastases in comparison to neighboring non-tumorous tissues demonstrated the specific accumulation of the pathological material in the core matrisome and several collagen-modifying enzymes including MMPs, A Disintegrin And Metalloproteinase (ADAM)s and Lysyl oxidase homolog 1 (LOXL1; Naba et al., 2014). Desmoplasia and disturbances of collagen are a characteristic of colorectal cancer, and several collagens, the most frequent of which are type I, VI, VII, VIII, X, XI, and XVIII, have been identified in colorectal cancer specimens collected (Skovbjerg et al., 2009; Kantola et al., 2014; Sole et al., 2014; Burke et al., 2015; Qiao et al., 2015; Shang et al., 2018; Yuzhalin et al., 2018b). The type I collagen has been seen to be elevated in cancerous tissues compared to normal tissues (Zou et al., 2013). In accordance with this finding, type I collagen mRNAs in the blood of colorectal cancer patients were also elevated over healthy individuals (Zou et al., 2013; Rodia et al., 2016). Consequently, second harmonic generation of fibrillar collagen content imaging has proven clinical usefulness in the stratification of high-grade cancers and its value in forecasting disease outcome in colorectal cancer patients (Birk et al., 2014; Burke et al., 2015).

**FIGURE 5:**
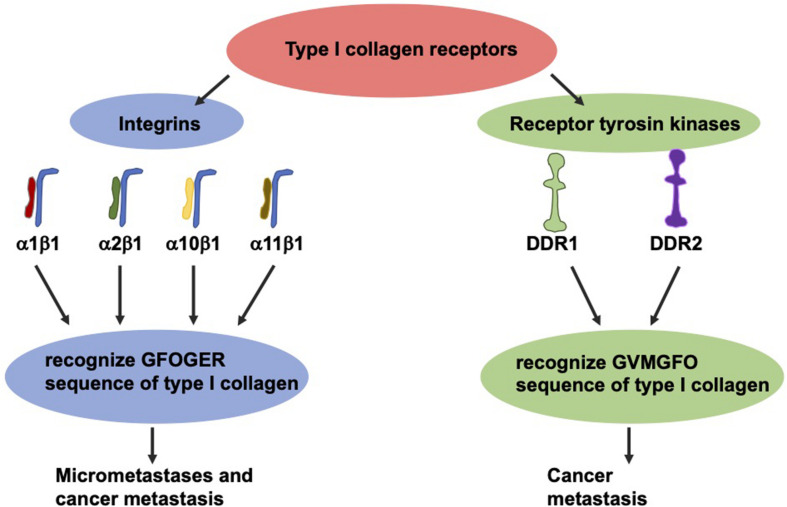
The two groups of type I collagen receptors, the integrins and the discoidin domain receptors. These two types recognize different sequence motifs in type I collagen.

The most thoroughly investigated type I collagen receptors are integrins α1β1, α2β1, α10β1, and α11β1 (Figure 5; Barczyk et al., 2009). After identification of their GFOGER sequence, the activation of these receptors can be switched on by various ligands such as collagen type I (Knight et al., 2000). Among these collagen receptors, the α1β1 integrin represents the most expressed one in colon carcinoma (Boudjadi et al., 2013). Moreover, the expression of the β1 integrin subunit in tumors correlates with diminished overall survival and decreased disease-free survival in a broad-ranged cohort of colorectal cancer patients (Liu et al., 2015). In specific, the β1 integrin is found in the serum of colorectal cancer patients, and the degree of its expression seems to strongly correspond with the levels of aggressiveness and the occurrence of micrometastases (Assent et al., 2015). The overexpression of the β1 integrin is tightly coupled with the malignant progression of colorectal cancer and consequently ends up in the colorectal metastasis of the targeted organs, such as the liver (Sun et al., 2014; Assent et al., 2015). In line with this, the β1 integrin expression is decreased *in vitro* due to sensing of 3D type I collagen (Assent et al., 2015; Saby et al., 2016).

Collagen can also transduce signals to cells via two RTKs discoidin domain receptors (DDRs), referred to as DDR1 and DDR2 (Figure 5). Each of which are able to interfere with type I collagen (Rammal et al., 2016) and fulfill an essential part in the progression of the cancer disease (Saby et al., 2016). These receptors, featuring tyrosine kinase activity, identify the GVMGFO sequence of type I collagen (Konitsiotis et al., 2008) and undergo fairly tardy and persistent activation (Vogel et al., 1997). DDR1 is highly expressed in colon carcinoma and encourages the development of metastases in invasive colon carcinoma (Chen et al., 2011; Jeitany et al., 2018, Sirvent and Lafitte, 2018). With respect to DDR2, high levels of expression were linked to higher abundances of T4, lymph node metastases, peritoneal dissemination and a poor prognosis, indicating that DDR2 expression appears to be an efficacious therapeutic objective (Sasaki et al., 2017).

### Hydrogel Gradients

A technique for the fabrication of type I collagen hydrogels with precisely controlled and reproducible physical density and mechanical stiffness gradients by avoiding damage to the collagen fibril structure and omitting additional chemical alterations is highly needed (Kayal et al., 2020). In order to encourage the development of appropriate substrates, key knowledge must be gained about the influence of local stiffness gradients both in respect of the value of the principal stiffness and the slope of the gradient. A set of model substrates with collagen hydrogels was generated and characterized, in which predefined stiffness gradients were established and analyzed. Therefore, tissue engineering technology was adapted to generate gradients in RAFT-stabilized collagen hydrogels (Cheema and Brown, 2013; Levis et al., 2015) by constructing molds and 3D printing molds to obtain model collagen hydrogels with well specified stiffness gradients. These collagen constructs were employed to examine the impact of stiffness gradient and stiffness magnitude on the elongation and orientation of cells *in vitro* (Kayal et al., 2020).

A gradient hydrogel constitutes a hydrogel which exhibits a gradual and continuously spatio-temporal variation in at least one of its properties (Genzer and Bhat, 2008). Gradient hydrogels provide outstanding engineering instruments for the native-like, biomimetic cellular microenvironment. They also enable the determination of a broad range of property levels in a single specimen, which is ideal for high-throughput screenings (Sant et al., 2010). Gradients may be physical or biochemical or a mixture of the two and may also contain a time dimension. Gradient hydrogels can be produced by several techniques that typically entail the initiation of precursor solution crosslinking, such as photopolymerization, the enzyme-catalyzed technique and thermally initiated gradient (Vega et al., 2017). When crosslinking enhances stiffness, the first covered hydrogel area behaves predominantly elastic, whereas the last covered area, that has been subjected to UV light the longest, exhibits a higher rate of crosslinking and is the stiffest. Hence, a gradient of stiffness is generated (Hudalla and Murphy, 2015). Apart from UV-light cross-linking, specific enzymes including tyrosinases, transferases, and peroxidases can be utilized to catalyze covalent cross-linking of hydrogel precursors and subsequently to modify hydrogels (Teixeira et al., 2012).

Biochemical gradients are gradients in concentration of the bioactive molecules, such as morphogens, which encompass a broad variety of substances including growth and transcription factors, chemokines, and cytokines. Biocompatible hydrogels can be based on natural and some synthetic polymers, that exhibit inherent bioactive characteristics. The bioactivity of hydrogels can be increased through distinct functionalization, which covers covalent binding of peptides and proteins (Hesse et al., 2017) or exopolysaccharides (Rederstorff et al., 2017) to the polymers of the hydrogel. Moreover, the elevation of bioactivity can be gained through additional specific affinity binding, which is facilitated by incorporating specific collagen binding sequences to the envisioned peptide/protein to be easily attached and connected into the collagen-based hydrogel (Hesse et al., 2018). In general, synthetic hydrogels are functionalized in a way that they become better suited for cell adhesion through the incorporation of cell-adhesive ligands, such as RGD(S) peptides, which presents the key element of the adhesion of cells to fibronectin (Moreira Teixeira et al., 2014).

### Porous Hydrogels

Porous hydrogels can be manufactured in multiple ways: firstly by producing the gel matrix scaffold with completely encapsulated biodegradable units (Zhao et al., 2013), secondly, by the production of hydrogel fibers through electrospinning or 3D bioprinting (Murphy and Atala, 2014; Bracaglia et al., 2017), thirdly, by the implementation of porogens, such as polymer microspheres (Fan and Wang, 2017; Gupta et al., 2017), or fourthly by usage of 3D laser perforation (Ahrem et al., 2014). Under mechanical stress, hydrogel frameworks also have an elongation gradient; the surface hydrogel layer dampens more elongation than the middle and deep layers (Brady et al., 2017).

The multi-branched PEG-based gradient hydrogel stage acts as biomimetic cell niche with autonomously adjustable matrix stiffness and biochemical ligand density, such as the CRGDS peptide (Tong et al., 2016). Both gradients were implemented in a timed manner, such as firstly the mechanical gradient and secondly the chemical gradient. Thereby, a gradient of UV exposure can be applied over the precursor solvent or over hydrogels with well-defined mechanical gradients (Tong et al., 2016).

### Cell-Derived Matrix Cross-Linking Molecules

**FIGURE 6:**
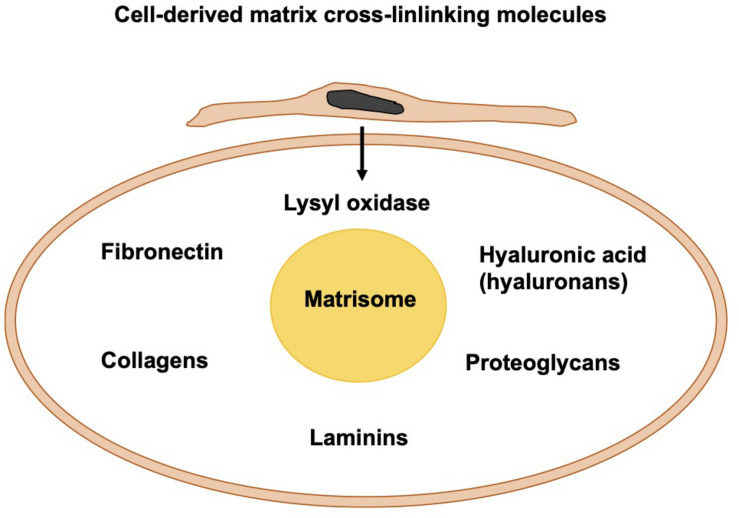
Cell-derived matrix cross-linking molecules create the matrisome.

Apart from the photo-crosslinked fibers, fibers can be crosslinked through biomolecules that are either cell-secreted or externally added to the migration and invasion assays. Among them are fibronectin, HA, and lysyl oxidase (Figure 6). In living tissues, the core of the matrisome comprises five different classes of macromolecules, namely collagens, fibronectin, hyaluronans, laminins, and proteoglycans. In the majority of tissues, fibrillar collagen is the primary source of extracellular matrix. Cells integrated into fibrillar collagen interfere with it via their surface receptors, such as integrins and DDRs. On one side, cells receive signals from the extracellular matrix that alter their functionalities and response. On the other side, all cells inside the tumor surroundings, for example, cancer cells, cancer-associated fibroblasts, endothelial cells, and immune cells produce and release matrix macromolecules under the regulation of several extracellular cues.

#### Fibronectin

An old classical focus of cancer research has been on the obvious changes in stiffness, as the near stroma of most tumors usually stiffens and thickens due to an accumulation of type I collagen and fibronectin (Miles and Sikes, 2014; Pickup et al., 2014). The factors that govern the balance between the deposition of the matrix and its degradation in the course of the disturbances of the tissue remodeling processes are required for enlightening the mechanisms that control a large set of normal and pathological processes. In fibronectin null cells, the polymerization of fibronectin into the extracellular matrix microenvironment is necessary for the accumulation of type I collagen and thrombospondin-1 and that the retention of fibronectin fibrils of the extracellular matrix demands the constant polymerization of a fibronectin matrix. Beyond that, the integrin ligation by itself is inadequate to sustain the extracellular matrix fibronectin in the complete lack of fibronectin accumulation. It has been shown that maintenance of thrombospondin-1 and collagen I in fibrillar constructions located inside the extracellular matrix relies on an effective fibronectin matrix. An undamaged fibronectin matrix is crucial for preserving the configuration of the cell–matrix adhesion sites. However, in total lack of fibronectin and fibronectin polymerization, neither α5β1 integrins nor tensin can attach to fibrillar cell–matrix adhesions (Sottile and Hocking, 2002).

The deposition of fibronectin into the extracellular matrix enhances the adhesion-dependent cell contractility (Hocking et al., 2000). Entire populations of cells are able to switch toward a dormant and stalled stage, which is induced by the assembly of an organized, fibrillar fibronectin matrix through cell–matrix adhesion via αvβ3 and α5β1 integrins, ROCK-generated tension, and the stimulation of TGFβ2 (Barney et al., 2020). The outgrowth of cancer cells after a dormancy period necessitates the MMP-2-driven breakdown of fibronectin (Barney et al., 2020). The impairment of the fibronectin polymerization facilitates a loss of collagen I matrix fibrils and an associated elevation in the amounts of endocytosed type I collagen (Shi et al., 2010). In addition, the membrane type matrix metalloproteinase 1 (MT1-MMP, synonymously referred to as MMP14) is critical for the regulation of the turnover of fibronectin (Shi and Sottile, 2011). The assembly of the matrix is generally triggered through extracellular matrix glycoproteins that connect to distinct cell surface receptors, such as dimerized fibronectin, which binds to α5β1 integrins. The binding of fibronectin to a receptor fosters the fibronectin self-association facilitated through the N-terminal assembly domain and primes in a structural manner the actin cytoskeleton to generate cellular contractility. Alterations in the conformational state of fibronectin lead to the liberation of additional binding sites, that contribute to the assembly of fibrils and consequently to their transformation into a stabilized and insoluble state. After their assembly, the fibronectin matrix governs the organization of tissues insofar that it manages the assembly of additional extracellular matrix proteins (Singh et al., 2010).

**FIGURE 7:**
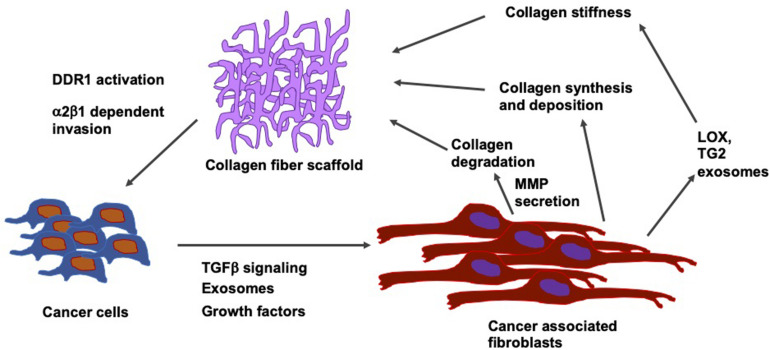
Relation between type I collagen fiber scaffolds and cells including their tumor microenvironment, such as cancer associated fibroblasts (CAFs). Cancer cells secrete TGF- β, growth factors and exosomes. CAFs influences collagen synthesis and deposit collagen and release LOX and transglutaminase 2 (TG2) exosome that subsequently increases the collagen stiffness.

In breast cancer, the stiffening of the tissue through the accumulation of fibronectin and collagen is linked to an advanced progression of the disease at both locations, the site of the primary tumor and metastatic sites (Libring et al., 2020). Intracellular and soluble fibronectin is at first lost during the transformation of the tumor, whereas it is restored in all lines exhibiting epithelial-mesenchymal plasticity. Non-transformed mammary epithelial cells cannot cause accumulation of fibronectin matrices except when transglutaminase 2, which functions as a fibronectin cross-linking enzyme, is overexpressed. On the contrary, breast cancer cells alter the fibronectin matrix output of fibroblasts in a phenotype-specific fashion. In addition, different levels of accumulation were found according to whether the fibroblasts are conditioned to replicate paracrine signaling or endocrine signaling events of the metastatic niche. In the paracrine signaling, fibroblasts stimulated with breast cancer cell cultures of high epithelial-mesenchymal plasticity have been shown to lead to the largest accumulation of the fibronectin matrix. In endocrine signaling, mesenchymal breast cancer cells generate extracellular vesicles, which cause the highest levels of matrix formation through conditioned fibroblasts. Consequently, it has been revealed that there exists a dynamic interplay between cancer cells and surrounding stromal cells inside the tumor microenvironment. During that interplay, the amount of fibronectin and its degree of fibrillarization within the extracellular matrix are adapted to the specific stage of progress of the disease (Figure 7). In contrast, in invasive cancers, these fibrils reorient themselves vertically to the boundary of the tumor and serve as so-called tracks for the migration of cancer cells across the basement membrane (Yang et al., 2011; Clark and Vignjevic, 2015; Bayer et al., 2019).

#### Hyaluronic Acid

Apart from fibronectin, the matrices can also be altered mechanically through HA. The adhesion/homing molecule CD44, participating in cell-cell and cell–matrix adhesion, constitutes the major cell surface receptor for HA (Lesley et al., 1992). The transcription machinery of YAP1/TAZ and TEA domain transcription factor (TEAD) activates the transcription of CD44 by coupling to the CD44 promoter at TEAD binding sites, thereby encouraging the propagation of malignant pleural mesothelioma (MPM) cell lines (Tanaka et al., 2017). Apart from CD44, the receptor for hyaluronic acid-mediated motility (RHAMM) synonymously referred to as HMMR, IHABP, or CD168, acts as a receptor for HA (Turley et al., 2002).

The aberrant expression of RHAMM that is generally not present in normal tissues, fosters the proliferation, migration and invasion of cells and consequently causes the resistance to pharmacological drugs in distinct types of tumors encompassing breast (Wang et al., 2014), lung (Wang et al., 2016) and liver cancers (He et al., 2015). Moreover, HA enhances the migration and invasion of cells in MPM cells via RHAMM (Shigeeda et al., 2017). Receptor for hyaluronic acid-mediated motility is transcriptionally regulated through Yes-associated protein 1 (YAP1) and transcriptional co-activator with PDZ-binding motif (TAZ), whose own expressions are found to be elevated in cancer cells during their malignant progression (McCarthy et al., 2018; Ye et al., 2020). It is obvious that YAP1/TAZ and TEAD complexes couple to a specific location within the RHAMM promoter and thus are capable of governing cell migration and invasion in breast cancer cell lines (Wang et al., 2014). In line with this, it has been shown that inhibitors, such as Verteprofin and CA3, both suppress the mesothelioma phenotype that encompasses cell migration and invasion, spheroid assembly and subsequently the formation of the primary tumor (Kandasamy et al., 2020). In specific, these two reagents cause apoptosis in mesothelioma cells. It is known that the persistently active YAP1 expression abolishes the action of the inhibitors, which leads to the hypothesis that impairment of the YAP1/TAZ/TEAD signaling is a prerequisite for the effectiveness of Verteporfin and CA3 in mesothelioma cells.

Under normal physiological circumstances, the stabilization and activation of YAP1/TAZ are closely guided through the phosphorylation within the Hippo pathway (Lei et al., 2008; Liu et al., 2010; Zhao et al., 2010). However, under pathological conditions, when this pathway is dysregulated, it has been revealed that this can lead to aberrant stabilization and activation of YAP1/TAZ protein, resulting in tumor development, progression, metastasis, and even recurrence (Ma et al., 2015; Zanconato et al., 2016). Moreover, by acquiring cancer stem cell-like characteristics, it can elicit a resistance to drugs (Lian et al., 2010; Cordenonsi et al., 2011; Touil et al., 2014; Zhao et al., 2014; Tanaka et al., 2017).

#### Lysyl Oxidase

The extracellular matrix can be seen as a vastly fluctuating system, which permanently undergoes a process of continuous conversion driven by the cells that populate it. As a consequence, neighboring cells are forced to adapt their behavior (Butcher et al., 2009). In the microenvironment of the tumor, abnormal extracellular matrix dynamical processes are prevalent and account for the course of progression, transformation and dispersal of cancer cells. One hallmark of cancer, for example, is the excessive production and secretion of extracellular matrix proteins encompassing collagen I, II, III, V, and IX, that all cause fibrosis of tissues (Zhu et al., 1995; Kauppila et al., 1998; Kalluri and Zeisberg, 2006; Huijbers et al., 2010; Pickup et al., 2014). The tissue fibrosis, in turn, raises the stiffness of the surrounding microenvironment of the tumor compared to the surrounding tissue, thereby accelerating the progression of the cancer by lowering the concentrations of the tumor suppressors PTEN and HOXA9 within the cancer cells (Mouw et al., 2014; Pickup et al., 2014). It has been revealed how breast adenocarcinoma cells release lysyl oxidase, a substance that crosslinks extracellular matrix proteins, resulting in secondary stiffening of the extracellular matrix scaffold to ease invasion (Levental et al., 2009). This rise in stiffness also affects the surrounding cells, including the development of cancer-associated fibroblasts (Calvo et al., 2013) and tumor-activated macrophages (Acerbi et al., 2015).

In identifying key features of the extracellular matrix to alleviate these transformations, the matrix mechanics of the tumor environment has an outstanding character (Kalluri and Weinberg, 2009). For instance, in the course of the epithelial to mesenchymal transition (EMT), where polarized epithelial cells are transferred toward more motile mesenchymal cells during biological processes including embryogenesis and the malignant progression of cancer (Chen et al., 2017), laminin-rich extracellular matrix is able to abolish EMT, whereas fibronectin-rich extracellular matrix fosters the transformation of it (Chen et al., 2013). Stiffening behavior of the surrounding microenvironment has also been reported to support the EMT of breast cancer cells that consequently enhances its invasive capacity and malignant progression encompassing cancer metastasis (Wie et al., 2015), whereby the polarity of tissues provides elevated resistance to death in mammary cancer cells (Weaver et al., 2002).

### Cell-Derived Matrix-Degrading Molecules

Another major focus of cancer research is the investigation of the matrix-degradation through cell secreted molecules such as MMPs, including MT-MMP1 (synonymously referred to as MMP-14) (Wolf et al., 2003, 2007; Sabeh et al., 2009) and MMP-2 (Clark and Weaver, 2008), whose substrates are collagen and fibronectin, as well as MMP-9 (Jacob and Prekeris, 2015), which all have demonstrated to fulfill a major role in the malignant progression of the tumor. In colorectal cancer, the co-culture of tumor associated macrophages and colorectal cancer cells elevates the generation of cancer-derived MMP-2 and MMP-9 (Kang et al., 2010).

The basement membrane surrounding tissues or vessels and the fibrillar collagen-based interstitial matrix represent physical barriers for cell migration and invasion (Willis et al., 2013). A dense, cross-linked extracellular matrix is mainly assembled by type I collagen (Hanahan and Weinberg, 2000; Chambers et al., 2002). Extracellular matrix molecules can also be broken down extracellularly through the employment of proteases such as MMPs, plasminogen activators, and plasmin (Hynes, 1990; Marchina and Barlati, 1996; Shapiro, 1998). In line with this, the dense extracellular matrix scaffold serves also as barrier and can be overcome in two different ways, one mechanical mode without the degradation of the extracellular matrix (Friedl et al., 2001; Wolf et al., 2003, 2007) and one enzymatic mode involving collagenolytic activity through MMPs, such as mainly MT1-MMP in the course of cancer invasion (Stetler-Stevenson and Yu, 2001; Itoh and Nagase, 2002; Hojilla et al., 2003; Cruz-Munoz et al., 2006).

Even though leukocytes (and amoeboid cancer cells, which travel through the extracellular matrix using hydrostatic pressure and membrane swellings referred to as blebs) apparently migrate protease-independently, mesenchymal cancer cells have to free their route through focusing breakdown activity. MT1-MMP can be considered a membrane-bound matrix metalloprotease that performs a distinct, non-redundant function in the invasion of a number of cancer cell types (Sabeh et al., 2009). In contrast, the principal protrusion of invasive cancer cells has the potential to attract and regulate the extracellular matrix fiber alignment (without large-scale degradation). An integrin-rich and actin-rich area of collagen decomposition has been identified, which lies behind it but still in front of the nucleus (Wolf et al., 2007).

The Arp2/3 activator N-WASP has been revealed to fulfill a crucial task in focal proteolytic decomposition of the extracellular matrix. The actin polymerization facilitated by N-WASP encourages the infiltration of MT1-MMP at actin hot spots, which are accumulations of F-actin at places of interaction and contact with the extracellular matrix. MT1-MMP is bound to these actin hotspot herds by means of an actin-binding domain located inside the cytoplasmic tail. Thus, N-WASP-facilitated actin polymerization guides protease activity by producing actin hotspots in immediate vicinity of matrix fibrils intended for breakdown in invasive cells (Yu et al., 2012). The WASP family member WASH fosters the Arp2/3-driven polymerization of actin on late endosomes, and provides tubules that are able to fuse with the plasma membrane at locations of FAs that provide cell matrix adhesion (Monteiro et al., 2013). Therefore, various Arp2/3 nucleation promoting factors, which operate at multiple subcellular sites, could combine to control a matrix degradation regime at areas of extracellular matrix contact to eliminate the extracellular matrix barrier and thereby ease the cell protrusion. In view of the fact that the size of the matrix pores is a major limitation for the transmigration of moving cells (Wolf et al., 2013), it is intriguing to assume that sites of cell-matrix contact in front of the nucleus could serve as a narrowing belt that is liberated by these focal proteolytic processes in invasive cancer cells.

**FIGURE 8:**
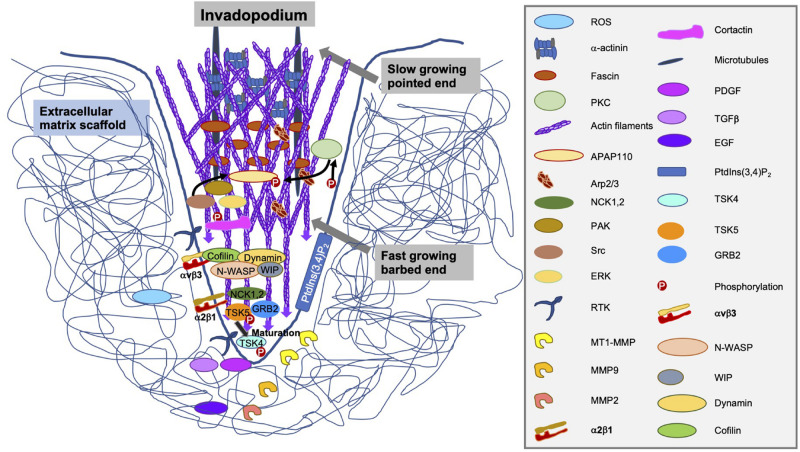
Structure, components and secreted enzymes of an invadopodium.

In the maturation phase of invadopodia, they accomplish to be proteolytically active, consisting of a process marked by the identification and/or secretion of specific functional MMP enzymes (Figure 8). The three members of the MMP family compromise MMP-2, MMP-9 and MT1-MMP that are linked within invadopodia (Jacob and Prekeris, 2015). Of special interest for this pivotal finding is MMP-2, which has fibronectin type II repeats that attach to its collagen substrate (Polette et al., 2004). MMP-2 is targeted on invadopodia, where it is released into the extracellular surroundings to breakdown the extracellular matrix (Clark and Weaver, 2008). Most interestingly, the overexpression of cofilin in multiple invasive cancer cell lines enhances the cellular invasiveness and increases the enzymatic activity of MMP-2 (Yap et al., 2005; Dang et al., 2006). In contrast, the reduction of cofilin expression lowers the maturation of invadopodia and subsequently, the enzymatic activity of MMP-2 (Wang et al., 2007; Tahtamouni et al., 2013).

A hypothesis takes on a necessary function for the membrane-tethered collagenase MT1-MMP through its capacity to proteolytically breakdown matrix components including type I collagen (Seiki, 2003; Sabeh et al., 2004; Fisher et al., 2006; Li et al., 2008). These results strongly indicate that MT1-MMP-expressing cancer cells have the intrinsic ability to move through the extracellular matrix scaffold according to the collagenolytic activity of this enzyme. However, clinical trials aiming these enzymes have revealed no promising outcomes (Coussens et al., 2002).

When cancer cells expressing MT1-MMP, such as HT1080s cells, are trapped in the dense physiologically networked type I collagen scaffolds, such as 2.0–5.0 g/l type I collagen, and broad-spectrum inhibitors of MMPs are supplemented, no MMP-independent invasion is reported (Sabeh et al., 2004; Fisher et al., 2006). Cells secrete metalloproteinases, including these MMPs, to breakdown the tight collagen matrix scaffold and enhance the pore size to encourage the invasion of cells. This phenomenon is more evident when the density of the fibers is high and the spaces between the fibers are considerably reduced compared to the size of the cell body (Fraley et al., 2015). Incorporating the activity of MMPs in the model leads to an enhancement of cell invasion at higher collagen concentrations, consistent with melanoma invasion data. The MMP-dependent reshaping of the fibers also offers the necessary room for cell proliferation in the close proximity of the cells. Consequently, the elimination of MMPs is proven to decrease cell proliferation (Bott et al., 2010). However, there is no one-way alteration, since a single alteration has usually side effects, which impacts migration and invasion, that has to be taken into account. The rigidity of the matrix also influences the proliferation of the cells in a Rho-based mode. Enhancement of matrix stiffness and Rho activity initiates phosphorylation of FAK at Y397 sites, which has been associated with an acceleration of cell migration and invasion (Mierke et al., 2017).

### Differences in Matrix Remodeling Mechanisms Between Normal and Malignant Cells

Metastasizing breast carcinoma cells can utilize normal mammary branching mechanisms during organ morphogenesis to elevate their tissue-invasive activity. The mechanisms of breast cancer invasion revealed that MT1-MMP fulfils a functional role, while the closely related proteinase MT2-MMP (synonymously referred to as MMP-15) does not appear to be associated, although it acts as a predominant proteolytic participant in bifurcation morphogenesis and the invasion of carcinoma cells *in vivo*. In contrast, the epithelial cell-specific aiming of MT1-MMP in normal mammary glands leaves the branching unaffected, while the loss of the proteinase in the carcinoma cells reverses the invasion, maintains the matrix structure, and totally prevents metastasis. In contrast, in the normal mammary gland, extracellular matrix reshaping and morphogenesis is only removed when MT1-MMP expression is specifically eliminated from the periductal stroma. These results reveal the complementary but diverse mechanisms that govern the developmental schemes compared to those for redesigning the neoplastic matrix (Feinberg et al., 2018).

Complicated signal transduction cues govern the cancer invasion in 3D extracellular matrices confinements, such as MMP-dependent and MMP-independent mechanisms. Lysophosphatidic-acid-triggered HT1080 cell invasion necessitates MT1-MMP-dependent collagenolysis to obtain matrix voids of nuclear width. These spaces are referred to as single-cell invasion tunnels (SCITs). When SCITs are generated, cells manage to migrate MMP-independent inside them. Apart from cancer cells, endothelial cells, smooth muscle cells and fibroblasts can produce SCITs during their invasion, which leads to the hypothesis that the generation of SCIT is a basic element of cellular motility inside 3D matrices. Precisely regulated signaling actions are necessary for the generation of SCITs. MT1-MMP, Cdc42 and its downstream effectors, including myotonic dystrophy kinase-related Cdc42-binding kinase (MRCK) and p21 protein-activated kinase 4 (PAK4), protein kinase Cα and Rho-associated coiled-coil-containing protein kinases (ROCK-1 and ROCK-2) guide the synchronization required for SCIT construction. Finally, MT1-MMP and Cdc42 are fundamental compounds of a cointegrated invasion signaling system that is capable of directing single cell invasion in 3D collagen matrices (Fisher et al., 2009).

## Second Direction (Extrinsic Mechanics-Based Approach): Mechano-Invasion Assays

**FIGURE 9:**
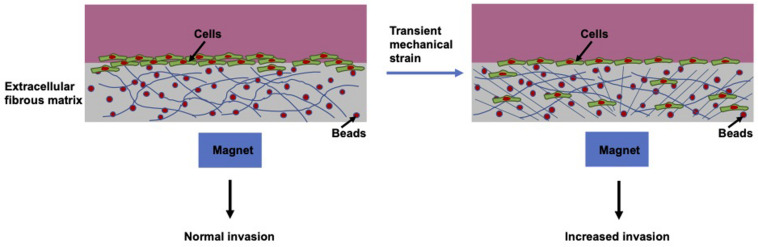
Schematic drawing of mechano-invasion assay.

Apart from the biochemically based metastatic cascade, with increasing understanding of the metastatic progression, there are also a number of mechanical parameters that favor the initiation of the multi-stage metastatic cascade (Kumar and Weaver, 2009). A broad range of mechanical forces are observed within the surrounding tissue environment of cancers. In contrast, an enormous amount of research has concentrated exclusively on the elevated rigidity of the tumor stroma (Paszek et al., 2005; Kostic et al., 2009; Levental et al., 2009). However, when employing an *in vitro* mechano-invasion assay (Menon and Beningo, 2011) the impact of various kinds of mechanical cues can be examined on the capacity of a cell to migrate and invade through matrix scaffolds. Mechanical stimulation takes the form of temporary tugging forces generated through magnetic beads coincidentally connected to anisotropic collagen and fibronectin fibers (Figure 9). These forces are not sufficiently strong to cause stretching of the entire material, nor is the transient strain oriented in a specific axis of the substrate.

In biomechanical research, a major focus is on alterations of the structure and mechanics of the entire tissue, and another major focus is on local biophysical alterations affecting the geometry and topology of the surrounding extracellular matrix. Primarily, an early focus has been on pronounced alterations in stiffness, as in most cases the tumor nearby stroma commonly becomes more rigid and dense that has been caused by an elevated generation of collagen type I and fibronectin (Miles and Sikes, 2014; Pickup et al., 2014). This enhancement in matrix rigidity may foster an elevation in cancer cell proliferation, migration and invasion (Alexander et al., 2008; Kostic et al., 2009; Parekh and Weaver, 2009; Ulrich et al., 2009; Tilghman et al., 2010; Charras and Sahai, 2014; Jerrell and Parekh, 2014; Umesh et al., 2014). Beyond the matrix rigidity, stroma-associated cells, such as the highly contractile myofibroblasts, generate forces within the extracellular matrix environment, when they remodel and invade through the extracellular matrix (Goffin et al., 2006; Shieh, 2011; Tripathi et al., 2012). In the course of the remodeling action of the matrix, a tugging force is produced and transferred toward the surrounding collagen fibers that are further bundled and rearranged (Goffin et al., 2006; Castella et al., 2010; Murrell et al., 2015; Oudin et al., 2016a). The occurrence of myofibroblasts and the forces they exert have been implicated in enhanced invasion and motility of cancer cells (De Wever et al., 2008; Elkabets et al., 2011; Fuyuhiro et al., 2012).

The transient mechanical strain can be evoked by embedding magnetic particles inside hydrogels, such as 3D collagen fiber matrices and exerting displacements on those beads with an external permanent magnet. This approach seems to be appropriate to mirror the magnitude and organization of the forces, which are caused by residual normal fibroblasts or fibrosarcoma cells, through transient tugging on fibers inside collagen–fibronectin matrices. Consequently, this approach increases the level of invasive cells, when they are *per se* highly metastatic cells, such as human fibrosarcoma cells (Menon and Beningo, 2011). This indicates that random transient tugging forces seem to deliver mechanical cues, which can be employed to foster the invasion of metastatic cancer cells.

These distinct mechanical cues and even more can be detected within the extracellular matrix through mechanoreceptors of the plasma membrane (Gasparski and Beningo, 2015). The integrin receptor family is involved in mechanoreception and its relevance in mechanotransduction has been extensively examined (Roca-Cusachs et al., 2012; Ross et al., 2013). It is generally known that cellular structures including filopodia, lamellipodia and invadosomes, encompassing invadopodia, and podosomes, react to mechanical stimuli (Schwarz and Gardel, 2012; Mrkonjic et al., 2016).

### Transient Mechanical Strain Affects Highly Invasive Cells

Transient mechanical strain facilitates maturation of invadopodia, elongation of invadopodia and subsequently a pronounced migration and invasion of cells into hydrogels due to this mechanical load (Menon and Beningo, 2011; Gasparski et al., 2017). Hence, enhanced invasion due to this mechanical stimulus necessitates the cells to be invasive by themselves, since non-invasive cells cannot be induced to be invasive. This mechanical signal is utilized especially by metastasizing cancer cells. Cofilin and fibronectin are required to react to mechanical stimuli. The expression of cofilin in cancer cells is required to maintain their invasive potential (Walter et al., 2009; Menon and Beningo, 2011; Nagai et al., 2011). Cofilin activity is governed through LIM kinase 1 (LIMK1) phosphorylation at its Ser3 position. Unphosphorylated cofilin interacts with F-actin filaments to trigger actin polymerization, whereas phosphorylated cofilin cannot fulfill this task. Consequently, exclusively active, unphosphorylated cofilin interferes with actin polymerization through the production of free barbed-ends (Blanchoin et al., 2000).

The integrin β3-encoding gene is specifically expressed, which confirmed its functional role in sensing this type of mechanical probing. The downregulation of integrin β3 expression raises the maturation of invadopodia due to stimulation, whereas a knocked down expression of cofilin creates invadopodia that are insensitive to stimulation. As a consequence of encouraging maturation of invadopodia, there is a concomitant enhancement of MMP activity levels linked to invadopodia. Tugging forces have been established as intrinsically supportive of metastatic cell invasion, and they continue to deliver a mechanical stimulus for the foundation of mechanically initiated maturation of invadopodia (Gasparski et al., 2017).

The integrin β3 forms pairs with integrin αv or integrin αIIb to create the heterodimeric integrin molecules. These integrin β3-containing heterodimers interact with arginine–glycine–aspartic acid (RGD) domains of fibronectin (Xiong et al., 2002). Since fibronectin is necessary for the enhanced reaction of cells in this kind of mechano-invasion experiments (Menon and Beningo, 2011), the decreased expression of integrin β3 by mechanical stimulation is fascinating. In addition, it is understood that the levels of expression of integrin β3 may differ in several cancer types and that this variation in expression can influence cell invasion (Jin and Varner, 2004; Sheldrake and Patterson, 2009; Seguin et al., 2015). When the downward regulation of the integrin β3 would be mandatory for the cells to perceive the mechanical stimulus and generate an increased invasion, overexpression of integrin β3 would impede the amplification of the invasion. In fact, it has been shown that cells overexpressing integrin β3 cannot react to mechanical stimulation, as they did not exhibit increased invasion compared to controls, indicating that downregulation of integrin β3 is necessary for increased invasion as a consequence of stimulation.

### Interaction Between Transient Strain and Expression of Proteins

Multiple cases of integrin crosstalk have been documented in which the attachment of one integrin to its ligand results in a disturbance in the expression or activity of other integrins. This type of crosstalk exists between integrin β3 and another fibronectin-binding family member, integrin β1 (Gonzalez et al., 2010). However, there is no crosstalk between integrin β1 and β3 subunits in the invasive reaction due to mechanical stimulation (Gasparski et al., 2017). Is there a relationship between integrin β3 subunits and cofilin? Cofilin severs actin filaments and matures invadopodia (Yamaguchi et al., 2005). In the mechano-invasion assay, cells treated with cofilin siRNA cannot sense the mechanical stimulation and exhibit no increased invasion, instead they displayed a basal invasiveness (Menon and Beningo, 2011). Since cofilin activity is governed by its phosphorylation at Ser3, which is referred to as Ser3 phospho-cofilin (Pollard and Borisy, 2003), it is supposed that mechanically triggered cells exhibit decreased amounts of Ser3 phospho-cofilin (inactive form) compared to non-stimulated controls. Moreover, when cofilin activity relies on integrin β3 expression, overexpression of integrin β3 is expected to elevate the amount of inactive phospho-cofilin due to mechanical strain. It has been observed that levels of Ser3 phospho-cofilin are reduced due to mechanical stimulation, which leads to the hypothesis that more cofilin needs to be in its active state after mechanical stimulation. This elevation in the amount of active cofilin is driven through decreased expression of the integrin β3 mechanoreceptor (Gasparski et al., 2017).

Cofilin activity facilitated actin polymerization is necessary for invadopodia maturation. Thus, knockdown of cofilin is expected to impair the elongation of invadopodia triggered by mechanical stimulation of cells. In line with this, MMP-2 represents a hallmark of invadopodia-based invasion. But it has been found that HT1080 cells have an MMP-independent nuclear piston invasion mechanism that demands integrin β3 activity (Petrie et al., 2017). It is a fascinating concept that a cell employs the expression of integrin β3 to choose between various invasion modes (Mierke, 2019b, 2020), so that down-regulation favors MMP-dependent invasion, whereas the normal integrin levels of β3 expression foster an MMP-independent nuclear piston invasion mode. The actin filament-severing activity of cofilin is controlled through LIMK1-dependent phosphorylation (Arber et al., 1998; Pollard and Borisy, 2003; Yamaguchi et al., 2005). Apparently, there exists a model signal transduction pathway that combines integrin β3 and cofilin activity and subsequently the ripening of invadopodia. It has been proposed that the engagement and activation of integrins causes the activation of Rac1, a Rho-GTPase regulated through integrin β3 within focal complexes (Morgan et al., 2009). Therefore, the hypothesis is that mechanical stimulation decreases the expression of integrin β3, which in turn decreases the activation of Rac1. In opposition to other investigations, in which Rac1 expression strengthened the migration and invasion of cells (Kunschmann et al., 2019). It is hypothesized that various types of force use distinct signal transduction pathways, in which the force is primarily modulated by the interaction of cells with various ligands.

It has been determined that an increased stiffness of the invasion assay matrix (4.5 g/l) had no effect on the increase of invasion through tugging forces compared to a less stiff matrix (2.5 g/l). Besides, an increase in stiffness only, without the tugging force, has not raised the invasion level (Menon and Beningo, 2011). However, this is in contrast to other studies where stiffness *per se* may increase the invasiveness of another metastatic cancer cell line, the MDA-MB-231 breast cancer cells, or even normal MCF10A epithelial cells (Fischer et al., 2017, 2020). However, it may be that different cell types respond differently to stiffness changes. Following the first finding, it can be assumed that highly invasive cells are able to distinguish a tugging force from an alteration in matrix stiffness and interpret the signal in a different way, which supports the hypothesis of differential sensing of forces within extracellular matrices or tissues. This type of mechanical signaling may exist in every extracellular microenvironment and is obviously utilized by the heavily metastatic cancer cells to encourage invasiveness, whereas non-invasive cells do not evidently have the ability to exploit this special type of mechanical signature.

#### Role of PAK in Mechano-Invasion

The p21-activated kinase 1 (PAK1) belongs to the six-member PAK serine-threonine-protein kinase family. It encompasses three major domains: firstly, a kinase domain in the C-terminus, secondly, an autoinhibitory domain and thirdly, a p21-binding domain (Kumar et al., 2017; Rane and Minden, 2018). The auto-inhibitory domain of PAK abolishes the catalytic activity of its own kinase domain. A single PAK1 molecule is normally inactive, but is activated when its auto-inhibitory domain connects to the kinase domain of another molecule (Kumar et al., 2017). PAK1 governs cytoskeletal remodeling, cell migration and invasion, metastasis and angiogenesis (Hammer et al., 2013; Hammer and Diakonova, 2015; Kumar and Li, 2016). The PAK family crucially connects Rho family of GTPases with several cytoskeletal processes. For instance, Rac1 interacts with PAK1 signal transduction in cell migration and invasion (Meyer Zum Büschenfelde et al., 2018; Mierke et al., 2020). Significant findings also suggest that PAK1 is implicated in numerous types of cancer, in particular in the control of the capability of invasive cells to metastasize (Hammer et al., 2013; Hammer and Diakonova, 2015; Yang et al., 2015; Kumar and Li, 2016).

In fact, a decrease in integrin β3 signaling due to mechanical stimuli, such as transient tugging force, is connected to the activity of PAK1 (Gasparski et al., 2019). It is well established that PAK1 exhibits a reduced activity under mechanical challenge, as evidenced by a reduction in Ser144 phosphorylation. However, this phosphorylation deficit can be restored when integrin β3 is overexpressed. PAK1 mutants display a coordinated reaction in the expression and activity of the MMP-2 enzyme, in addition to prolongation of invadopodia, as a reaction to stimulation. These findings have led to the recognition of a novel mechanosensitive reaction in human fibrosarcoma involving PAK1 as a signal generator which is downstream of integrin β3. There is an established cascading pathway linking integrin β3 to the control of cofilin activity, although its part in this mechanosensitive process is largely unclear. Integrin β3 transmits signals to Rac1 that result in the activation of PAK1 at the membrane through PAK1 autophosphorylation (Morgan et al., 2009). PAK1 can phosphorylate LIMK1 at the Tyr507 position, which decreases cofilin activity through phosphorylation of cofilin at its Ser3 position (del Pozo et al., 2000; Pollard and Borisy, 2003). Nevertheless, it is not established whether this route is of any relevance for the statement that transient mechanical stimulation leads to an elevation of cofilin activity and consequently to an intensified invasion through the maturation of the invadopodia. Most probably, this route could be scaled down, as this would generate more active, unphosphorylated cofilin, resulting in the maturation of invadopodia.

In human fibrosarcoma cells, the expression of PAK1 is reduced and also its phosphorylation (decreased phospho-Ser144 levels) due to transient stimulation. When integrin β3 is overexpressed, phospho-PAK1 levels are elevated in stimulated cells, indicating that PAK1 is in fact more active. When mutants of PAK1 were expressed in these cells, the so-called kinase-dead mutants displayed enhanced cell invasion, invadopodia maturation and related MMP-2 release. On the contrary, constitutively active PAK1 mutants exhibit a lower invasion, smaller invadopodia and reduced MMP-2 activity. These findings indicate that a reduction in PAK1 activity is required for fibrosarcoma cells to increase their invasiveness as a response to mechanical excitation.

### Mechanical Cues Induce Directed Motility

Directed cell movement, such as cancer stem cells, provided by contact guidance within aligned collagenous extracellular matrices represents a crucial point for the dissemination of breast cancers throughout tissues (Ray et al., 2017). The physical microenvironment impacts multiple fundamental cellular functions encompassing the migration of cells (van Helvert et al., 2018). Cell migration can be directed through the rigidity of the microenvironment by employing a process referred to as durotaxis (Lo et al., 2000). Durotaxis, migration to increasing stiffness, is involved in physiological and pathological mechanisms ranging from developmental processes (Flanagan et al., 2002; Sundararaghavan et al., 2009) to malignant progression of cancer (Butcher et al., 2009; Levental et al., 2009; Ulrich et al., 2009; Lachowski et al., 2017). Durotaxis necessitates cells to be able of firstly sensing a mechanical cue (mechanosensing), secondly directing their motility toward the mechanical stimulus (referred to as mechanically directed motility due to anisotropic mechanics), and thirdly migrating in a directed manner to the stimulus. These processes are critical for durotaxis, however, the molecular mechanisms governing them are mainly elusive.

Cells can react to mechanical requirements of the local microenvironment by changing their actin cytoskeleton in a dynamic fashion at FAs (Choquet et al., 1997; Butcher et al., 2009). In accordance with such findings, mathematical and experimental simulations pointed to the fact that the acto-myosin cytoskeleton in FAs provides an alternating traction force necessary for mechanically guided motility, and the directional motility toward a mechanical impulse (Plotnikov et al., 2012; Wu et al., 2017). Nevertheless, the mechanisms that govern these FA cytoskeletal dynamics, and the special part that these mechanisms perform as mechanosensors, in mechanically directed motility, and in durotaxis, still need to be unraveled.

The Ena/VASP family member Ena/VASPlike (EVL) is a new type of regulator of actin polymerization in FAs, and EVL-facilitated actin polymerization has been identified to control cell matrix adhesion and mechanosensors. In addition, EVL plays a decisive part in managing the mechanically directed motility of normal and cancer cells, encourages durotactic invasion and, intriguingly, the inhibition of myosin contractility does not hamper this mechanism. Importantly, suppression of EVL expression interferes with the durotactic 3D invasion of cancer cells. In extension, the reaction to chemotactic (biochemical) stimulation is amplified in cells with decreased EVL expression, indicating that EVL uniquely facilitates the reaction to mechanical stimuli. A scheme is put forward in which EVL-driven FA actin polymerization strengthens FAs while mechanically paced, which thereby improves mechanosensors, mechanically oriented motility and durotaxis (Puleo et al., 2019).

The expression of MENA or VASP failed to re-establish the diminished adhesion phenotypes in EVL KD cells, and by using chimeric mutants it was determined that the EVH1 domain of EVL is singular amongst the Ena/VASP proteins and is particularly relevant for the proper operation of EVL in FAs. These observational findings reinforce the growing evidence that Ena/VASP proteins, formerly assumed to be exchangeable in functional terms (Laurent et al., 1999), fulfill a singular and distinctive function in the regularity of FAs. For this purpose, MENA, for instance, singularly attaches to the integrin α5 and acts as a modulator of adhesion signal transduction through an actin-independent machinery (Gupton et al., 2012), and VASP works with the rap-1 interacting molecule (RIAM) and zyxin to govern the fluctuations of the integrin β1 (Worth et al., 2010) and the intactness of actin stress fibers (Smith et al., 2010). Thus, whereas data establish EVL as the principal Ena/VASP protein in charge of actin polymerization-based cell–matrix adhesion, MENA and VASP contribute substantially, but inevitably, to FAs. Most significantly, prior investigations have demonstrated that MENA has a critical importance in fostering chemotaxis and haptotaxis (Goswami et al., 2009; Oudin et al., 2016a,b), which are both regimes of directed cell migration that rely on soluble or fixed ligand gradients. Consequently, these findings support the hypothesis that Ena/VASP proteins are involved in a variety of tasks in directed cell migration and invasion. The important thing is that these proteins could probably be instrumental in incorporating biochemical and mechanical cues from the cell microenvironment to manage migration in physiological and pathological frameworks.

## Third Direction (Cell Mechanics-Based Approach): Cell Mechanics Are Altered to Regulate Cell Migration and Invasion

Dissimilar to intracellular proteins, which are persistently replaced (Jennissen, 1995), extracellular matrix proteins are extraordinary long-lived proteins (Shapiro et al., 1991; Sivan et al., 2006). Therefore, it is very likely that the cell mechanical properties will be altered to enable the migration and invasion of the cells. In concrete terms, the mechanical performance of animal cells is governed by a matrix of stiff protein filaments referred to as the cytoskeleton. The cytoskeleton is a noteworthy piece of material that is kept out of balance by a multitude of molecular mechanisms involving chemical energy (Mackintosh and Schmidt, 2010). The molecular motors that harness the energy generated during ATP hydrolysis to travel alongside actin filaments and microtubules constitute an integral part of this process (Howard, 2001). A strong evidence exists that the myosin II motors interacting with the actin filaments can actively enhance cell stiffness by producing a contractile prestress (Wang et al., 2001; Fernandez et al., 2006; Gallet et al., 2009; Kollmannsberger et al., 2011; Crow et al., 2012). Measurements with pure actin networks have demonstrated that these meshes become severely stiffened when either an external or an internal stress is imposed (Mizuno et al., 2007; Koenderink et al., 2009). Cells can leverage this non-linear stress answer to rapidly adjust their stiffness in the wake of extracellular environment stiffness alterations (Solon et al., 2007; Mitrossilis et al., 2010).

As fibrin gels stiffen strongly when probed by an external load, it can be proposed that active cell contraction enhances the elastic modulus of fibrin gels (Storm et al., 2005; Brown et al., 2009; Piechocka et al., 2010). Therefore, the linear elastic modulus, G_0_, of fibrinogen solutions including or excluding cells during its polymerization has been determined through macroscopic shear rheology. When cells are absent, G_0_ immediately raises and becomes a plateau after roughly 3 h. This increase reflects the rapid fibrin polymerization to a space-filling elastic scaffolding to be followed by a more retarded process of covalent crosslinking by FXIIIa (Standeven et al., 2007; Münster et al., 2013). In the presence of cells, an immediate elevation of G_0_ occurs, however, 4 h are required to obtain a constant G_0_ value. In specific, the rise of G_0_ has been revealed to be biphasic, with a bending around 2 h. The first phase of scaffolding stiffening probably resembles the same scaffolding formation process that arises without cells. The second phase of scaffolding stiffening roughly concurs with the beginning of cell spreading (Jansen et al., 2013). Finally, the entire network is shifted toward a stress-stiffened regime.

**FIGURE 10:**
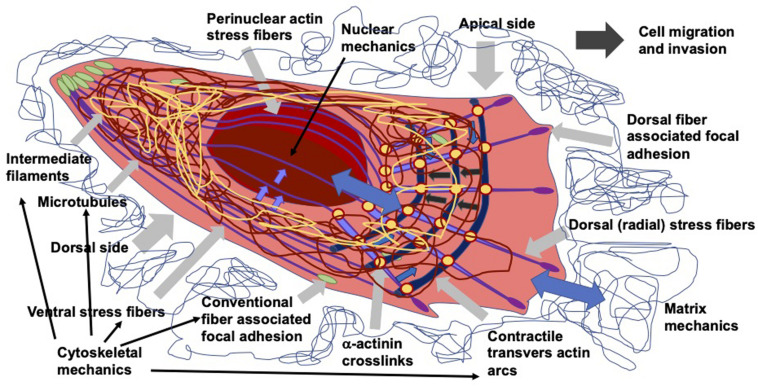
Interaction between the cell’s cytoskeletal mechanics, nuclear mechanics and the matrix mechanics impacts cell migration and invasion through environmental confinements.

In the majority of studies, the term cell mechanics has been employed to explore the effect of mechanical alterations inside cells on their microenvironment and even on the cellular functions, such as cell migration and invasion (Figure 10). In the following, the parts of the distinct cellular compartments, such as nucleus and other major organelles, or major cellular structures, such as actin filaments and myosin filaments, are briefly highlighted.

### Cytoskeletal Mechanics

A major contributor of cytoskeletal mechanics are actin filaments. The actin cytoskeleton is composed of semi-flexible actin filaments, myosin motors, and cross-linking proteins. *De novo* actin polymerization seems to be crucial for the generation of actin fibers in migrating cells (Hotulainen and Lappalainen, 2006), whereas accumulation of existing actin filament fragments is most probable in stationary cells in a steady-state surrounding (Tojkander et al., 2012).

During the adaptation procedure, the actin cytoskeleton reshapes itself to better withstand the external stress (Salbreux et al., 2012; Blanchoin et al., 2014; Mueller et al., 2017). In this way, actomyosin networks cross-linked by α-actinin and other cross-linking proteins are capable of adjusting to external forces through rapid mechanical reactions, in which stress relaxation appears on the time axis of seconds (Mizuno et al., 2007; Kasza et al., 2010; Schmoller et al., 2010; Dasanayake et al., 2011). However, cytoskeletal responses such as the (de)polymerization of actin or the activation of myosin II, where chemical energy is progressively transferred into mechanical force, results in delayed transformation of the actomyosin scaffolds on a time axis of minutes (Besser and Schwarz, 2007,; Ni and Papoian, 2019). As a consequence of the myosin-dominant mechanochemical dynamism, actomyosin networks are prone to contraction (Levayer and Lecuit, 2012; Floyd et al., 2019).

Apart from actin filament polymerization based protrusive forces, it has been hypothesized that the suppression of myosin contractility has an impact on the directionality of cell movement (Puleo et al., 2019). There is an upregulation of genes/proteins such as Ubiquitin domain−containing protein 1 (UBTD 1) that interact with E2 enzymes of the ubiquitin–proteasome system due to environmental cues, such as stiff matrices (Torrino et al., 2019). Rather unexpected is the fact that suppression of myosin activity had no impact on mechanically guided directed motility. Myosin contractility is involved in single-cell (Raab et al., 2012) and collective cell durotaxis (Sunyer et al., 2016) and commonly in cell migration (Vallenius, 2013). Nevertheless, no clear role of myosin in the process of perceiving directed mechanical stimuli has been documented. Even though it influences long-distance migration, the perception of directional impulses can be managed by various molecular processes (Mouneimne et al., 2004, 2006).

Significantly, the repression of myosin contractility disturbed the contraction of the back of the cell when motility was mechanically aligned, which is in agreement with previous findings (Sunyer et al., 2016). Consequently, myosin may be superfluous for targeting the cells to a mechanical stimulus (mechanically directed motility), but it is nevertheless essential for long-distance migration toward enhanced stiffness (durotaxis). Due to this complexity, future work is required to completely characterize the specific function of myosin in each of these distinct mechanically governed processes. Moreover, it has turned out that microtubules and intermediate filaments account for cell mechanics in a similar manner as actin filaments, which needs also to be taken into account.

### Nuclear and Other Organelle Mechanics

Since the nucleus is the largest and stiffest organelle within a cell (2- to 10-fold stiffer as the neighboring cytoplasm), it represents a major obstacle for the migration and invasion of cells through narrow subcellular-sized constrictions (Lammerding, 2011; Liu et al., 2014; Mierke, 2017; Krause et al., 2019). The positioning and transport of the nucleus is performed by the coordinated actions of protrusion of the leading edge, integrin adhesion to the ambient environment, contractile actin cytoskeleton fibers, microtubules and the linker of the cytoskeleton and nucleoskeleton (LINC) complex that physically couples the chromatin to the cytoskeleton. This leads to a fluctuating force accumulation at the front and a contraction of the back while removing the rear integrin from the extracellular matrix, thereby displacing the nucleus to the front. Accordingly, during cell migration, alternating pulling and pushing cycles operate on the cell nucleus, resulting in what is known as the multi-stage translocation cycle of the cell nucleus (Friedl et al., 2011; Wolf et al., 2013; Wu et al., 2014; Thomas et al., 2015; Jayo et al., 2016; McGregor et al., 2016).

The steps comprise firstly the exertion of pressure on the nuclear membrane in the direction of migration due to the external constriction, secondly the incipient deformation of the nucleus due to the formation of a local incident retarding the migration, thirdly the sliding of the compressed and deformed nucleus through the pore and fourthly the backward release, combined with a fast forward pushing and rounding (recoil) of the nucleus (Friedl et al., 2011). Hence, the migration and invasion are retarded in the second step, which is consistent with the physical barrier role of the nucleus and seems to be a storage phase of deformation energy that is released as propulsive energy during the fourth phase enabling elevated movement.

Subsequently, the nucleus is rate-limiting for the migration of cells through subcellular pores or 3D extracellular matrix scaffolds (Jia et al., 2005; Wolf et al., 2007; Beadle et al., 2008; Friedl et al., 2011). The shape and the size of the nuclei can be quite diverse due to the specific cell type and distinct stimulation or treatment of cells. The entire stiffness of the nucleus of intact cells relies on several structural determinants, encompassing A-type lamins being a component of the nuclear lamin network underneath nuclear membrane and the organization of chromatin (Pajerowski et al., 2007; Swift et al., 2013; Stephens et al., 2017).

The chromatin packing state changes continuously depending on transcriptional requirements, the level of DNA repair and the specific cell cycle phase. Any of these events bring about modifications in the DNA organization, with temporary and reversible transformations from dense heterochromatin to more open euchromatin due to histone acetylation or demethylation (Sarma and Reinberg, 2005; Hizume et al., 2010; Unnikrishnan et al., 2010) that consequently cause chromatin decondensation, nuclear softening and ultimately its enlargement (Toth et al., 2004; Chalut et al., 2012).

Despite of this, the majority of cells exhibit in 3D substrates rather egg-shaped or spherical shaped nuclei with a diameter between 5 and 15 μm (Jaalouk and Lammerding, 2009). Under extracellular matrix confinement close to the nuclear diameter, or in the presence of cell-derived proteolytic tissue degradation activity generating a low resistance migratory path that resembles close to the largest cell diameter, the migration and invasion is not hindered (Wolf et al., 2013). In contrary, in an ambient constriction by an extracellular matrix, the cell nucleus has to deform and adjust to the matrix confinements, while simultaneously constituting a mechanical impediment that gradually retards the migration and invasion (Fu et al., 2012; Wolf et al., 2013; McGregor et al., 2016; Thiam et al., 2016).

The nuclear mechanics have been shown to be regulated by cytoskeletal components, such as actin filaments (Fischer et al., 2020). Beyond the nucleus, the mechanical properties of other organelles, such as mitochondria, Golgi apparatus or endoplasmic reticulum can impact the overall mechano-phenotype and thereby impact the behavior of cells, such as cell migration and invasion.

## Future Direction of Cell Mechanics Research Facilitating Motility

Combined approaches of the three different directions seem to be promising to gain new insights into the mechanical processes and how matrix mechanics, cell mechanics and nuclear and other organelle mechanics are interrelated. There is generally a bidirectional interaction between cells and the surrounding extracellular matrix with a special focus on the mechanical phenotypes of cells and matrices. The ultimate goal seems to be still on tunable mechanical cues applied to 3D engineered matrices with varied biochemical and embedded cell compositions. Another focus of future research will lie on the co-culture approaches, where the surrounding cells, such as cancer associated fibroblasts or macrophages and cancer associated endothelial cells can foster or impair the migration and invasion of cancer cells by possibly affecting mechanical phenotype of cancer cells, including the cytoskeletal as well as the nuclear and other organelle phenotype, or the mechano-phenotype of extracellular matrices.

## Author Contributions

CTM wrote the manuscript and prepared all the figures.

## Conflict of Interest

The authors declare that the research was conducted in the absence of any commercial or financial relationships that could be construed as a potential conflict of interest.
